# Therapeutic significance of tumor microenvironment in cholangiocarcinoma: focus on tumor-infiltrating T lymphocytes

**DOI:** 10.37349/etat.2023.00199

**Published:** 2023-12-28

**Authors:** Chaoqun Li, Lei Bie, Muhua Chen, Jieer Ying

**Affiliations:** Kumamoto University, Japan; ^1^Department of Hepato-Pancreato-Biliary & Gastric Medical Oncology, Zhejiang Cancer Hospital, Hangzhou Institute of Medicine (HIM), Chinese Academy of Sciences, Hangzhou 310022, Zhejiang, China; ^2^Postgraduate training base Alliance of Wenzhou Medical University (Zhejiang Cancer Hospital), Hangzhou 310022, Zhejiang, China; ^3^Department of Thoracic Surgery, Wuhan No.1 Hospital, Tongji Medical College, Huazhong University of Science and Technology, Wuhan 430030, Hubei, China

**Keywords:** Tumor microenvironment, cholangiocarcinoma, tumor-infiltrating T lymphocytes, molecular pathogenesis, immunotherapy

## Abstract

Cholangiocarcinoma (CCA) is a highly aggressive type of adenocarcinoma distinguished by its invasiveness. Depending on specific anatomical positioning within the biliary tree, CCA can be categorized into intrahepatic CCA (ICCA), perihilar CCA (pCCA) and distal CCA (dCCA). In recent years, there has been a significant increase in the global prevalence of CCA. Unfortunately, many CCA patients are diagnosed at an advanced stage, which makes surgical resection impossible. Although systemic chemotherapy is frequently used as the primary treatment for advanced or recurrent CCA, its effectiveness is relatively low. Therefore, immunotherapy has emerged as a promising avenue for advancing cancer treatment research. CCA exhibits a complex immune environment within the stromal tumor microenvironment (TME), comprising a multifaceted immune landscape and a tumor-reactive stroma. A deeper understanding of this complex TME is indispensable for identifying potential therapeutic targets. Thus, targeting tumor immune microenvironment holds promise as an effective therapeutic strategy.

## Introduction

**Figure 1:**
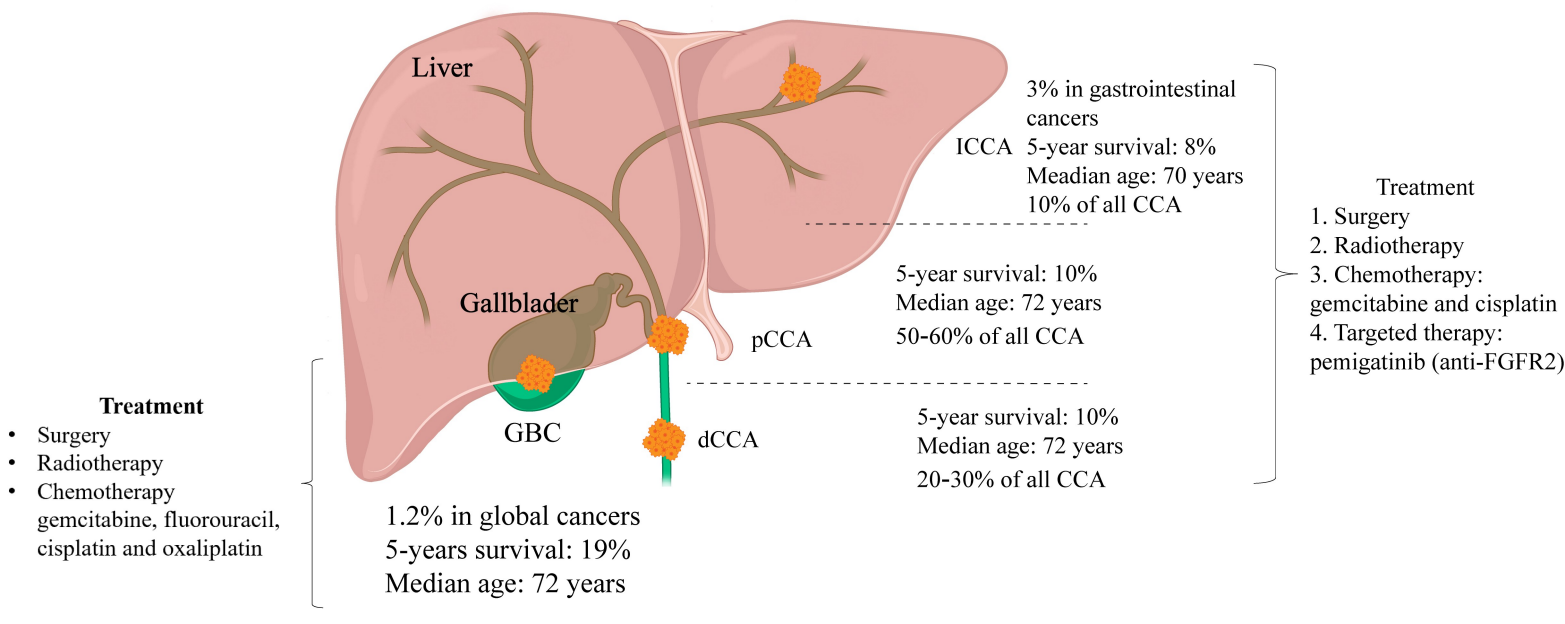
Based on the relative anatomical location within the liver, CCA can be categorized into three types: ICCA, perihilar CCA (pCCA), and dCCA. ICCA is characterized by bile duct cancer within the liver, with histological subtypes including mass-forming, periductal infiltrating, and intraductal growing; pCCA refers to a secondary branch located in the hepatic duct, specifically from the common bile duct above the cystic duct to the liver, and is histologically classified as a klatskin tumor; dCCA denotes located outside the liver, primarily presenting as adenocarcinoma histologically. GBC: gallbladder cancer; FGFR2: fibroblast growth factor receptor 2

Bile duct cancer, also known as cholangiocarcinoma (CCA), is a rare disease in which malignant cells form in the bile ducts that connect the liver and gallbladder to the small intestines (Figure 1). CCA is characterized by a complex etiology, late clinical diagnosis, high heterogeneity in anatomical location, immune microenvironment composition, and molecular variations [1]. Unfortunately, a rapid increase has been observed in the global incidence of one of the subtypes of CCA, intrahepatic CCA (ICCA) [2]. In addition, deep intrahepatic location, prominent fibrosis, low tumor cellularity characteristic, and highly heterogeneous and complex microenvironment to CCA, drastically reduce the chance of early detection and diagnosis of CCA [3]. Further, the progression of CCA is facilitated by cytokine secretion and chronic inflammation, as evidenced by previous studies [4–6]. Despite advancements in medical research, treatment options for CCA have been still limited to surgical intervention, chemotherapy, and radiation therapy. Currently, the first-line chemotherapy regimen for advanced or recurrent CCA is gemcitabine plus cisplatin. However, the efficacy of chemotherapy for CCA is low compared to other cancers [3, 7], which triggered the exploration of potential immunotherapies. The assessment standard of the immunotherapy for CCA includes Response Evaluation Criteria in Solid Tumors 1.1 (RECIST 1.1) and immunotherapeutic RECIST (iRECIST) trial guidelines. The immune-modified RECIST (imRECIST) has gained significant popularity in the management of intrahepatic lesions through locoregional therapy. However, it is important to note that imRECIST may not accurately capture atypical immune responses. For example, an increase in lesion diameter may be misinterpreted as “progression” resulting from locoregional immune activity. Consequently, the need to ascertain more specific molecular targets and efficacious treatment methods for CCA is becoming more urgent. With a greater number of molecular targets being identified, particularly with regard to the tumor microenvironment (TME) in CCA treatment selection, immunotherapy has emerged as a promising avenue.

Inflammatory cell infiltration may be employed as an endogenous biomarker for differentiating lymphocyte-infiltrated tumors from non-lymphocyte-infiltrated tumors. The immune cell population within lymphocyte-infiltrated tumors can exert either a promotive or inhibitory effect on tumor progression through immune responses [8, 9]. In CCA, T cells constitute the predominant subset of tumor-infiltrating lymphocytes (TILs) [10]. As the primary effector cells of the immune system, T lymphocytes are capable of recognizing and killing tumor cells by targeting tumor-associated antigens. These antigens may be tumor-specific neoantigens arising from somatic mutations within the tumor or overexpressed antigens [11]. Detection of tumor antigens by the immune system cells can be increased through inhibiting proteins on the surface of immune cells, referred to as immune checkpoint inhibitors (ICIs). Cancer cells exploit immune checkpoints to evade recognition by the immune system. Quite similarly, chimeric antigen receptor T-lymphocytes (CAR T) therapy consists of genetic modification of antigen receptors on the surface of a patient’s own T cells so that they recognize specific proteins or antigens found on the surface of cancer cells. Unsurprisingly, great progress has been made in both ICIs therapy and CAR T cell therapy. However, an understanding of characteristics and functionality of immune cells infiltration remains incomplete, thereby impeding development of immunotherapy. The immunotherapy strategies for CCA predominantly comprise adoptive transfer of engineered immune cells and immune checkpoint-targeted therapy. Consequently, the TME warrants further investigation [12, 13]. Numerous studies have documented the correlation between T lymphocytes and survival in patients with various solid tumors. In addition, tumor cells overexpress immune checkpoint molecules that subsequently release “don’t eat me” signals to immune cells. In this way, the checkpoint molecules establish an immune-suppressive TME that facilitates evasion of immune surveillance [14, 15]. It is noteworthy that the initial findings of the TOPAZ-1 trial indicate a significant improvement in overall survival (OS) and progression-free survival (PFS) for patients with advanced CCA when the checkpoint inhibitor, durvalumab is added to gemcitabine-cisplatin (GemCis). This improvement was achieved with an acceptable safety profile compared to the use of placebo plus GemCis. The findings regarding the efficacy of the treatment combining durvalumab with first-line chemotherapy regimens in advanced CCA are promising. They indicate that in the imminent future durvalumab in combination with GemCis may become a novel first-line standard therapy for CCA [16]. This review focuses primarily on the influence of infiltrating T lymphocytes on CCA progression.

## Immune landscape in CCA

**Figure 2:**
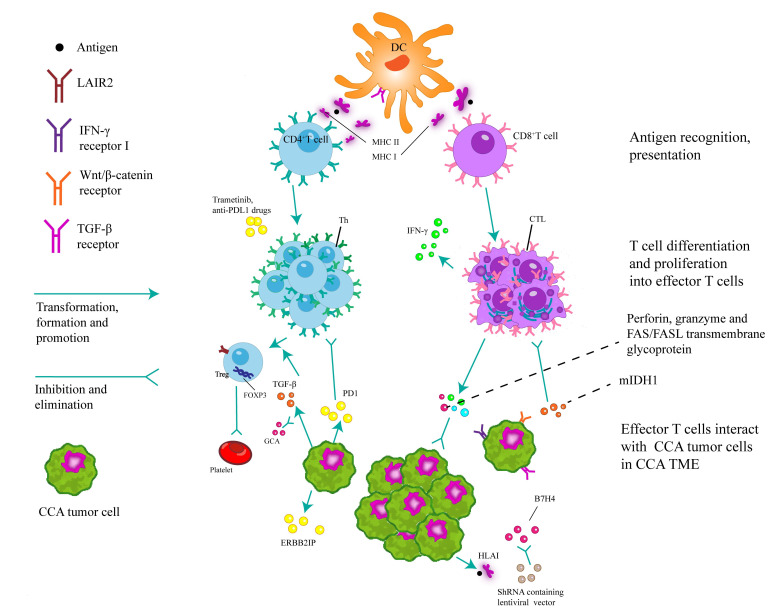
Dendritic cells (DCs) play a pivotal role in the activation of naive T cells through the process of antigen presentation via phagocytosis. Moreover, they are responsible for initiating immune responses, secreting chemotactic cytokines to attract T/B cells, and presenting antigens to CD8^+^/CD4^+^ T cells via major histocompatibility complex class I/II (MHC I/II) cells. Upon activation of CD8^+^ T cells, the release of perforin, granzymes and Fas cell surface death receptor (FAS)/Fas ligand (FASL) transmembrane glycoproteins lead to the eradication of tumors. The recruitment of CD8^+^ T cells and the expression of interferon-gamma (IFN-γ) were found to be hindered by inhibiting mutant isocitrate dehydrogenase 1 (mIDH1). Simultaneously, the expression of human leukocyte antigen class I (HLA I) facilitates the presentation of tumor antigen-derived peptides to the immune system, thereby eliciting anti-tumor effects through the activation of CD8^+^ T cells. The application of lentiviral transcription encoding short hairpin RNA (shRNA) to inhibit B7 homolog 4 (B7H4) has the potential to enhance CD8^+^ T-cell-mediated cytotoxicity. The potential of CD4^+^ T cells to elicit a response against the mutated ERBB2 interacting protein (ERBB2IP) antigen holds promise for inducing degeneration in metastatic epithelial cell carcinoma tissues. *In vitro* studies have shown that trametinib can upregulate of MHC I and programmed cell death ligand-1 (PDL1) on tumor cells. Furthermore, the combination of trametinib with anti-PDL1 drugs can increase the anti-tumor activity of hepatic effector memory CD4^+^ T cells. The upregulation of transforming growth factor beta 1 (TGF-β1) in neoplastic cells induces heterogeneity in regulatory T cells (Tregs) within the TME, which forms a milieu for the proliferation of tumor cells, inhibits apoptosis, and stimulates angiogenesis. This process ultimately facilitates tumor progression, but can be counteracted by the concurrent administration of gemcitabine in combination with carboplatin. The expression of leukocyte-associated immunoglobulin-like receptor-2 (LAIR2) by Tregs hinders the binding of LAIR1 to competing ligands, disrupts platelet activation and adhesion, and impedes the classical pathway of the complement system and the lectin pathway. As a result, LAIR2 inhibits the elimination of pathogens. CTLs: cytotoxic T lymphocytes; PD1: programmed cell death protein-1; Th: T helper

The intricate nature of the immune microenvironment in CCA contributes to inconsistent responses to anti-tumor treatments observed in patients [17]. A deeper understanding of the heterogeneity of immune infiltrates within CCA tumors can help develop personalized anti-tumor immunotherapies. In biliary tract cancer (BTC), the presence of intratumoral CD4^+^, CD8^+^, and forkhead box protein 3 (FOXP3^+^) T cells, which are predominant inflammatory cells, is associated with the OS of BTC patients [18]. The immunogenomic heterogeneity observed among CCA patients presents both opportunities and challenges for the development of personalized immunotherapies [19]. Based on hematoxylin and eosin (HE) staining, single-cell RNA sequencing, and a range of *in vitro* and *in vivo* analyses, the subpopulation of immune cells in CCA was classified into three groups, IG1 (immune-suppressive), IG2 (immune-rejecting), and IG3 (immune-activating) [20]. These immune subgroups have unique clinical, genetic, and molecular characteristics that are strongly correlated with the prognosis [20]. Using whole-exome sequencing (WES), RNA sequencing, T cell receptor (TCR) sequencing, and multiplex immunofluorescence techniques, Lin et al. [21] categorized ICCA into three subgroups: sparse, heterogeneous, and highly infiltrated. Additionally, they categorized ICCA into a high immune evasion group and a low immune evasion group, with distinct prognoses [21]. Better understanding of the roles of various immune cells in BTC and their interplay with CCA cells can facilitate the development of more efficacious therapeutic approaches (Figure 2).

### CD4^+^ T lymphocytes in CCA

CD4^+^ T lymphocytes play an essential role in regulating the immune system and promoting anti-tumor responses through interactions restricted by MHC II [22]. In contact with various antigens, naive CD4^+^ T cells can differentiate into distinct subsets, including Tregs, Th1, Th2, Th17, and follicular helper T cells. These subsets of CD4^+^ T cells produce a variety of cytokines that modulate inflammatory responses and activate gene programs in CD8^+^ T cells, thereby enhancing their anti-tumor response [23–25]. CD4^+^ CTLs represent a distinct subset of CD4 cells that exhibit cytotoxic activity. Both preclinical and clinical investigations have established the direct anti-tumor effects of CD4^+^ on CTLs by the release of cytotoxic granules containing granzyme B and perforin [26–28]. In the context of CCA, the overall quantity of tumor-infiltrating Tregs and CD4^+^ CTLs serves as independent prognostic markers for tumor grading and the Union for International Cancer Control (UICC) stage [18].

### Proportions and distribution of CD4^+^ T cells in CCA

Distribution of CD4^+^ T cells in the peritumoral and intratumoral regions of CCA has been explored by numerous studies; however, their findings remain conflicting. Specifically, five studies have reported a significant increase of CD4^+^ T-cell infiltration in the peritumoral region, compared to the tumor center in ICCA [18, 29–32]. Conversely, one study has demonstrated that CD4^+^ T-cell infiltration at the tumor edge is significantly higher than in the tumor center in both ICCA and extrahepatic CCA (eCCA) [18]. The BTC tumor edge serves as the primary location for active infiltration by FOXP3^+^CD4^+^ Tregs that express elevated levels of lymphocyte activation gene 3 (*LAG-3*) and T cell immunoglobulin and mucin domain-containing protein 3 (*TIM3*) [33, 34]. Furthermore, a high density of FOXP3^–^CD4^+^ Th cells at the tumor edge is independently associated with longer PFS and OS in patients undergoing palliative gemcitabine plus cisplatin treatment [33]. Conversely, another study has found no significant difference in the distribution of CD4^+^ T cells between the peritumoral and intratumoral regions [35]. The correlation between the distribution of inflammatory cells in CCA and local microvessel density (MVD) has also been established. Compared with metastatic CCA patients, immune cells, such as CD4^+^ T lymphocytes, of patients with locally advanced tumors are more infiltrated. Additionally, patients with metastatic CCA exhibit a significant increase in MVD, compared to those with localized CCA, indicating a negative correlation between MVD and inflammatory cell infiltration [36]. A further investigation demonstrated that a reduced MVD was associated with a poorer prognosis in patients undergoing treatment for ICCA. This was indicated by the significantly shorter recurrence-free survival observed in low MVD patients than in their high MVD counterparts. The correlation between MVD and CD8^+^ TILs infiltration was highly positive, while it was negative between MVD and FOXP3^+^ TILs, which could be attributed to the modulation of TILs recruitment [37].

#### Subset: CD4^+^ Tregs

Tregs have different functions than the FOXP3^–^CD4^+^ T-cell subset that is involved in supporting anti-tumor activities. Tregs exhibit the presence of CD4^+^CD25^+^FOXP3^+^ or CD4^+^CD25^+^CD127^low^ on their cell surface. Tregs possess immunosuppressive characteristics, thus regulate the immune response and help maintain immune homeostasis within the organism [38]. In relation to the spatial allocation of FOXP3^+^ TILs within the peritumoral and intratumoral regions of CCA, Goeppert et al. [18] discovered that these lymphocytes directly infiltrate the tumor mass in both ICCA and eCCA subtypes. Conversely, another study suggested that FOXP3^+^ TILs primarily reside in the peritumoral region [33]. One study has ascertained no discernible difference in the distribution of FOXP3^+^ TILs between the intratumoral and peritumoral regions [39]. Subsequent research has associated the presence of combined tumor protein p53 (*TP53*) and kirsten rat sarcoma vral proto-oncogene (*KRAS*) mutations with the accumulation of Tregs in ICCA [40]. In their study, Alvisi et al*.* [41] employed high-dimensional single-cell RNA sequencing, genomics, and cytometry to identify a substantial and significantly activated CD4^+^ Treg population within the tumor tissue of ICCA patients. This population was found to be closely correlated with the presence of the transcription factor mesenchyme homeobox 1 (*MEOX1*) in tumor cells. Moreover, the researchers discovered that targeted enrichment of *MEOX1* can induce a reprogramming effect on circulating Tregs, leading them to acquire the transcriptional and epigenetic characteristics observed in tumor-infiltrating Tregs. This finding highlights the potential role of *MEOX1* in modifying the functional properties of Tregs in the TME [41].

### CD8^+^ T lymphocytes in CCA

CD8^+^ T lymphocytes infiltrate in the TME to eliminate cancer cells [42]. These lymphocytes proliferate and, activated by a specific antigen, differentiate into effector cells that are specifically referred to as CD8^+^ cytotoxic CTLs. Retaining their killing function, CD8^+^ CTLs target and eliminate cells displaying a specific antigen through two distinct mechanisms: the perforin/granzyme pathway and the FAS/FASL pathway. On the former pathway, CD8^+^ CTLs induce apoptosis of target cells by releasing cytotoxic molecules, including perforin and granzyme B as well as cytokines. On the latter pathway, they induce apoptosis by binding the FASL to the FAS receptor on the target cell. The quantity and efficacy of CD8^+^ T cells significantly influence the anti-neoplastic response and thus the prognosis of patients with CCA [37, 43].

#### Proportions and distribution of CD8^+^ T cells in CCA

In comparison to hepatocellular carcinoma (HCC), CCA has a lower overall count of CD8^+^ T cells. However, CD8^+^ T lymphocytes prevail among the infiltrating inflammatory cells in CCA. The presence and positioning of CD8^+^ T cells within the tumor site have been associated with the clinical diagnosis and prognosis. Numerous studies have consistently demonstrated that CD8^+^ CTLs are predominantly localized in the peritumoral region of CCA, regardless of whether the CCA is ICCA or eCCA [18, 29–32]. If CD8^+^ T cells are expressing specific molecules, such as PD1 or CD103, they are predominantly localized within the tumor nests of ICCA [44, 45]. Conversely, a study has reported no clear disparity in the distribution of CD8^+^ CTLs between the peritumoral and intratumoral regions of CCA [31]. Treg cells exert a negative regulatory effect on the immune response by secreting immune-inhibitory cytokines, including interleukin-10 (IL-10) and TGF-β, which impede the activation and proliferation of CD8^+^ T cells [46]. Using The Cancer Immunome Atlas (TCIA) database, an examination of histological data from an internal cohort revealed that a high ratio of FOXP3^+^/CD8^+^ is a valid indicator of an unfavorable prognosis following curative resection of ICCA [47].

## Current state, recent advances and challenges of immunotherapy in CCA

**Figure 3:**
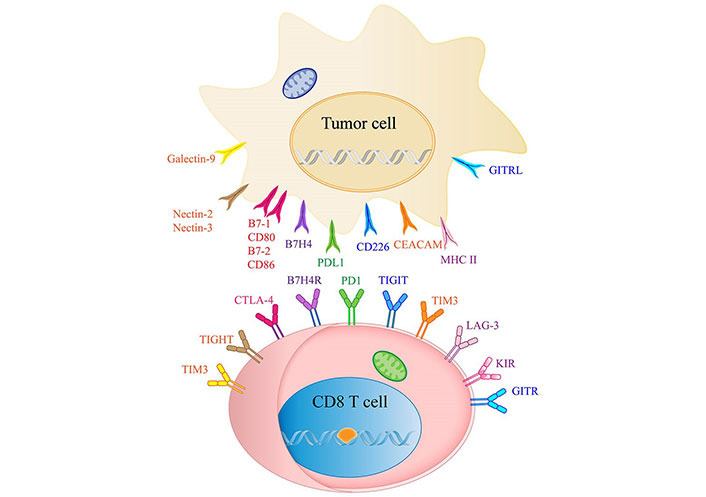
List of ICIs and their receptors in CCA. CEACAM: the carcinoembryonic antigen-related adhesion molecules; KIR: killer cell immunoglobulin like receptor; GITR: Glucocorticoid-Induced TNF-related protein; GITRL: GITR ligand; B7H4R: B7H4 receptor

The effectiveness of existing immunotherapies is frequently constrained by T-cell exhaustion [48]. It is a common phenomenon in which a majority of CD8^+^ T cells lose their functions due to prolonged exposure to antigens and the tumor immunosuppressive environment (Figure 3). These factors impede the ability of CD8^+^ T cells to mount an effective anti-tumor response [49, 50]. CD8^+^ CTLs express immune checkpoint molecules that limit their cytotoxic capabilities against CCA [51, 52]. In the realm of immune cells, several immune checkpoints have been identified. They include PD1, B and T lymphocyte attenuator (BTLA), cytotoxic T-lymphocyte associated protein 4 (CTLA-4), T-cell immunoglobulin and *ITIM* domain (*TIGIT*), *TIM3*, and *LAG-3* [53]. Nevertheless, dependable immune checkpoint markers would enable quick and accurate prediction of the therapeutic response in CCA treatment [54].

In a particular study, ICCA showed elevated levels of parameters associated with the response to ICI. A strong correlation was observed between a high proportion of tumor-infiltrating CD8^+^PD1^+^ T cells and the advanced tumor-node-metastasis (TNM) stage. Furthermore, patients with a higher CD8^+^/PD1 ratio experienced shorter postoperative survival periods compared to patients with a lower CD8^+^/PD1 ratio [30]. Thus, the CD8^+^/PD1 ratio served as an independent prognostic factor. The recent study found that N6-methyladenosine (m6A) demethylase AlkB homolog 5 (*ALKBH5*) interacts with PDL1 mRNA, leading to the inhibition of m6 modification in the 3’ untranslated regions (3’UTR) region of m6PDL1 mRNA, and promoting PDL1 expression in ICCA [55]. Upregulation of PDL1 suppresses T-cell expansion and cytotoxicity in ICCA [55]. A comprehensive analysis of a database and immunohistochemistry of tumor tissues from a cohort of 290 ICCA patients revealed that CTLA-4^+^ TILs and PDL1^+^ TILs independently predict tumor recurrence and OS in iCCA patients after surgical tumor removal [56]. Consequently, a therapeutic approach involving the concurrent targeting of PD1/PDL1 and CTLA-4 holds potential advantages for the management of ICCA patients.

ICCA has been found to exhibit the presence of inhibitory immune receptors. Transcriptomic analysis of exhausted T cells in a mouse model of primary liver cancer allowed to obtain valuable information on the expression of genes involved in the immune response and thereby on one of the proteins they encode, called *TIGIT*. TIGIT can accurately identify exhausted CD8^+^ T cells at various stages of differentiation. Furthermore, TIGIT is a prospective therapeutic target as it has been observed on tumor-infiltrating CD8^+^ T cells in samples obtained from patients with HCC and ICCA [57]. Jing et al. [58] found that, in ICCA the frequency of human endogenous retrovirus-H long terminal repeat- associating protein 2 (*HHLA2*) expression is higher than that of PDL1. Overexpression of HHLA2 is associated with a lower density of CD3^+^ TILs, CD8^+^ TILs, and a higher ratio of CD4^+^FOXP3^+^/CD8^+^ TILs. In contrast to PDL1, HHLA2 has been identified as an independent prognostic marker for OS in two separate cohorts [58]. Another study demonstrated a significant upregulation of B7H4 in ICCA compared to peritumoral tissues [59]. As a negative regulator of T cells, the immune checkpoint molecule *B7H4* induces epithelial-mesenchymal transition in ICCA, thereby facilitating tumor cell invasion and metastasis through the activation of the extracellular signal-regulated kinase 1/2 (ERK1/2) signaling pathway. Notably, patients with elevated *B7H4* expression in ICCA exhibit a reduced OS and PFS. Furthermore, the analysis of tumor samples has revealed a strong correlation between high *B7H4* expression and lymph node metastasis, advanced tumor stage, and poor differentiation. The expression of *B7H4* may be an independent prognostic indicator for the prediction of OS and tumor recurrence following surgical intervention in patients with ICCA [59]. Moreover, it is a potent candidate for a therapeutic target for individuals with this form of cancer. The analysis of clinical specimens obtained from 33 ICCA patients demonstrated that tissue-resident memory (TRM)-like CD8^+^ TILs expressing CD69^+^CD103^+^ exhibited significantly elevated levels of T-cell markers. Then, ICCA with lower infiltration of CD69^+^CD103^+^CD8^+^ TILs showed significant enrichment of genes associated with the Wnt/β-catenin and *TGF-β* pathways [45]. Consequently, this investigation posits that targeting CD69^+^CD103^+^ TRM-like CD8^+^ TILs could become an effective therapeutic intervention for patients with ICCA.

Multiple co-expressions on T cells indicate that combination therapy targeting various immune checkpoints may yield more effective therapeutic outcomes than a single-agent therapy focused solely on blocking PD1 [53].

### PD1/PDL1 expression

PD1/PDL1 has emerged as a prevalent clinical biomarker for prognosticating the effectiveness of immunotherapy in solid tumors [60]. To note, extensive clinical investigations have been conducted to assess the expression of PD1/PDL1 and its correlation with the response to ICI therapy in CCA. Nevertheless, unlike other solid tumors, the predictive value of PDL1 in CCA remains inconclusive based on existing studies. In the KEYNOTE-158 study, it was observed that the objective response rate (ORR) among patients with positive PDL1 expression was merely 6.6%, whereas the ORR for patients lacking PDL1 expression was 2.9% [61]. This finding suggests a modest association between PDL1 expression and the response to ICI. The findings from KEYNOTE-158 and KEYNOTE-028 trials indicate that the effectiveness of pembrolizumab therapy appears to be unaffected by the expression of PDL1 in CCA cells [62]. Interestingly, the detection rate of PDL1 also varied depending on the clone type, heterogeneity of samples, and limitations in sample size [19]. Specifically, studies using clone streptavidin-perosidase (SP) 142 reported a PDL1 positivity rate of 8.6%, whereas studies employing 5H1 (an PDL1 antibody to quantify PDL1 in tumors and immune cells) reported a rate of 72% [63]. Additionally, a study using E1L3 (an PDL1 antibody to quantify PDL1 in tumors and immune cells) revealed PDL1 expression in 30.5% of cancer cases [64]. Conversely, a recent clinical trial (NCT02829918) demonstrated that the clinical effectiveness of nivolumab was contingent upon the expression of PDL1 [65].

### Molecular pathogenesis of CCA related to TILs

Many signaling pathways, including Wnt/β-catenin, TGF signaling pathway, (an inhibitor of atypical protein kinase C-i) aPKC-i/phosphorylated transcription factor Sp1 (P-Sp1)/snail family transcriptional repressor (Snail) signaling pathway, hippo pathway, B7H1/PD1 pathway, and FAS/FASL pathway, are implicated in the malignant potential of CCA, as well as in immune evasion by inducing apoptosis of TILs [66–73]. Furthermore, apart from immune checkpoints, there is a growing research emphasis on exploring other molecular markers as potential targets for immunotherapy in this particular cancer type. Martin-Serrano et al. [40] conducted a transcriptomic analysis on a cohort of 900 ICCA cases by integrating components of the stroma, tumor, and immune microenvironment. As a result, they proposed a novel classification for ICCA termed the “stromal interaction molecule (STIM)” classification. Over 60% of ICCA cases exhibited a non-inflammatory phenotype, characterized by the presence of stemness-related pathways, BRCA1 associated protein 1 (*BAP1*), and isocitrate dehydrogenase 1 (*IDH1*) mutations, as well as focal loss of Salvador family WW domain containing protein 1 (*SAV1*). This phenotype is characterized by an abundance of immune inhibitory components [40].

### Yes-associated protein 1

It has been reported that cholangiocarcinogenesis is facilitated by Yes-associated protein (*YAP*) through transcriptional enhanced associate domain (*TEAD*)-dependent transcriptional activation of β-catenin, where the YAP interacts with the transcription factor TEAD to regulate gene expression [74]. In ICCA, β-catenin forms a binding association with YAP and is essential for the complete transcriptional activity of YAP, thereby highlighting the functional interplay between the YAP and β-catenin pathways in cholangiocarcinogenesis [70]. Song et al. [75] reported that focal adhesion kinase (FAK) activation induces the proto-oncogene YAP, contributing to ICCA initiation and progression. FAK inhibitors, alone or in combination with anti-cyclin-dependent kinase 4/6 (anti-CDK4/6) inhibitors, may be effective treatments for ICCA [75]. Since YAP1 stimulates the transformation of hepatocytes into ICCA, it is necessary to investigate the association between the YAP1 pathway and the immune inhibitory microenvironment in CCA [76].

### Mucin 1

In a recent study, Zhang et al. [77] identified the pivotal gene Mucin 1 (*MUC*1) in the progression of CCA and predicted its potential downstream carcinogenic pathways through high-throughput sequencing datasets from the Gene Expression Omnibus (GEO) database. As a result of the interaction between MUC1 and a cell surface receptor, epidermal growth factor receptor (*EGFR*), the *EGFR*/phosphatidylinositol 3-kinase (*PI3K*)/protein kinase B (*Akt*) signaling pathway is activated, resulting in the accumulation of FOXP3^+^ Treg cells. *In vivo*, this accumulation of cells enhances the malignant phenotype of CCA cells, which ultimately facilitates metastasis and promotes CCA growth [77]. These studies offer prospective and valid options for the treatment of CCA.

### IDH1

IDH1 and IDH2 are frequently mutated metabolic genes in human cancers [78, 79]. IDH mutations are particularly prevalent in ICCA among epithelial malignancies [80]. The presence of mutant IDH (*mIDH*) in ICCA promotes its development through the production of 2-hydroxyglutarate (*2HG*) and the inhibition of hepatocyte nuclear factor 4 (*HNF-4*) [81]. The pharmacological inhibition of mIDH1 has shown promise in delaying the progression of mIDH1 ICCA. However, the activation of immune cells and stimulation of Treg cells ultimately lead to tumor progression [82, 83]. Using genetically engineered mouse models driven by mIDH1, researchers inactivated the ten-eleven translocation 2 gene (*TET2*) of DNA demethylase with 2HG produced by mutant IDH enzymes. Significantly, researchers discovered that the combination therapy of *mIDH1* inhibitor AG120 (an orally active inhibitor of *mIDH1* enzyme) and anti-CTLA-4 antibody induced substantial cell death and sustained tumor-specific T-cell responses in tumor-bearing mice [84]. These findings support further development of immunotherapy for patients with *mIDH1*-mutant ICCA.

### Other biomarkers

Circular RNAs (circRNAs) are also a biomarker of iCCA. Circular RNA HMGCS1-016 (circHMGCS1-016) promotes invasion of ICCA through the miR-1236-3p/CD73 and galectin 8 (GAL-8) pathway while downregulating CD8^+^ T-cell infiltration. Furthermore, circHMGCS1-016 holds promise as a novel potential biomarker for predicting resistance to PD1 inhibition in ICCA [85]. In addition, CD73 plays a role in promoting the proliferation, migration, invasion, and epithelial-mesenchymal transition of ICCA. Analysis of clinical samples from 259 resected ICCA cases reveal a significant association between high CD73 expression and an increased proportion of FOXP3^+^/CD8^+^ TILs and CD163^+^/CD68^+^ tumor-associated macrophages (TAMs) [86]. Moreover, an increased expression of LAIR2 was correlated with poorer patient survival, indicating the potential of LAIR2 as a marker for exhausted T-cell populations [87].

### Engineering T cells for adoptive therapy: CAR T

**Figure 4:**
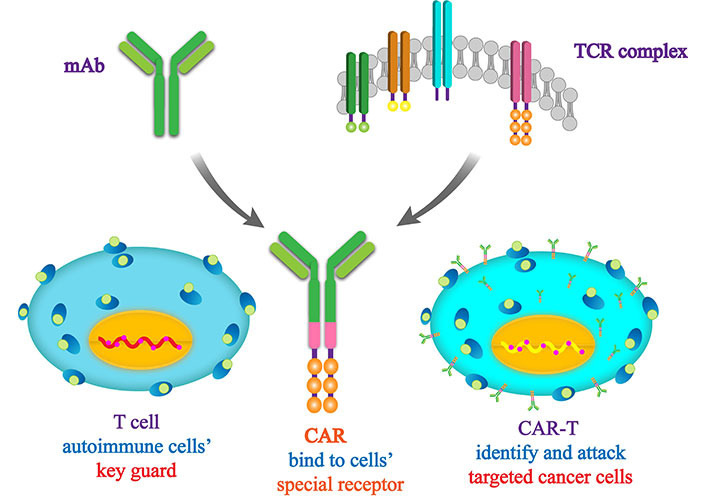
Structure of CAR T. mAb: monoclonal antibody

CAR T therapy is an immunotherapeutic approach that entails the genetic modification of T lymphocytes to express an artificial receptor capable of identifying and binding to particular target antigens (Figure 4). Extensive evidence has demonstrated the remarkable efficacy and long-lasting clinical responses of CAR T therapy in diverse hematological malignancies and solid tumors [88–92]. CAR T therapy, a breakthrough in cancer treatment, is currently being used to combat refractory CCA.

Human EGFR 2 (*HER2*)-CAR T have demonstrated high patient tolerability without dose-limiting toxicities [93]. Antigens such as *EGFR* and *B7H3* have been found to be highly expressed on CCA cells. Studies have shown that B7H3-CAR T and EGFR-CAR T possess specific antitumor activity. In addition, researchers have successfully engineered protein targeting to glycogen (PTG)-T16R-single chain fragment variable (scFv)-CAR T with immune checkpoint molecules such as *PD1*, *TIM3*, *TIGIT*, *TGF-β* receptor (T*GF-βR*), IL-10 receptor (IL-10R), and IL-6R knocked out. These hexavalent inhibitory molecule-knockout CAR T have demonstrated potent anti-CCA immunity and long-term efficacy both *in vitro* and *in vivo* [94]. Supimon et al*.* [95] have devised anti-MUC1-CAR (αM.CAR)/switch receptor (SR), which comprises a MUC1 scFv and a PD1-CD28 SR. These T cells have quickly eliminated CCA cells overexpressing MUC1, with partial mitigation of T-cell exhaustion induced by PD1 upregulation [96]. The fourth-generation CAR T were composed of an anti-MUC1 scFv and three co-stimulatory domains (CD28, CD137, and CD27) associated with CD3ζ. The study successfully demonstrated the specific cytotoxic activity of these CAR T against CCA cells [95]. Another study on antitumor efficacy in ICCA revealed the toxicity of Tn-MUC1 CAR T targeting glycosylated MUC1, both *in vitro* and *in vivo* [97]. In a recent study, fourth-generation A20-4G CAR T exhibited remarkable cytotoxicity against CCA cells expressing integrin integrin alphavbeta6/8 (αvβ6) [98]. Furthermore, T cells producing αCD133-αCD3 bispecific T-cell engagers (BiTEs) have been successfully engineered and thus have become highly specific against CD133-positive CCA [99].

CAR T therapy is used not only for primary CCA but also for secondary CCA. To address the unfavorable prognosis of metastatic CCA, researchers have adopted autologous infiltrating lymphocytes. The method has proved to be highly effective. In a particular study, the adoptive transfer of ERBB2IP-reactive CD4^+^ Th1 lymphocytes successfully mitigated the advancement of the disease [100]. White et al. [101] used whole exome and transcriptome sequencing to identify TCR-mediated, MHC II-restricted autologous CD4^+^ Th1 cells within TILs from a patient with metastatic CCA. These cells were able to recognize specific peptides that make up the FGFR2-tudor domain containing 1 (TDRD1) protein breakpoint in cancer cells. The studies presented empirical evidence of the ability of TILs from patients with invasive metastatic CCA to selectively identify peptides originating from FGFR2 fusions. Consequently, the findings of the aforementioned studies corroborate future investigations into adoptive transfer and fusion-reactive T-cell therapy, particularly for the treatment of fusion-driven solid tumors.

## Conclusions

Frequently asymptomatic until advanced stages and unresponsive to conventional therapies, CCA is a cancer with dismal prognosis. The substantial heterogeneity and intricate TME of CCA contribute to the ineffectiveness in achieving long-term survival. The progression and metastasis of CCA necessitate distinct oncogenic drivers and establish intricate communication networks within the TME, facilitating immune evasion and hastening tumor advancement. However, immunotherapy may be an effective response to this challenge. Presently, immunotherapeutic interventions for CCA include ICIs, cancer vaccines, and adoptive T-cell therapies. T lymphocytes hold significant potential as a viable target for CCA immunotherapy (Table 1). The distribution of infiltrating T lymphocyte subpopulations in CCA plays a significant role in tumor progression and the response to immune therapy. Moreover, extensive studies on T-cell exhaustion enable the development of immunotherapies that utilize ICIs and enhance the effectiveness of CD8^+^ T cells in CCA. The advancement of precise and targeted CAR T therapies presents a promising and individualized strategy for cellular immunotherapy in the context of CCA. Currently, combined therapies, including ICIs with chemotherapy, adoptive cell therapy, and tyrosine kinase inhibitors (TKIs), are being more actively investigated than monotherapies. Additionally, the combination of ICIs with topical therapies has generated considerable interest. Ongoing clinical trials aim to validate the effectiveness and safety of these combination strategies. In essence, TILs may serve as a significant prognostic indicator and potential therapeutic target. However, a deeper understanding the significance of TILs in CCA warrants further clinical and translational investigations. The research to date on TILs in CCA offers novel prospects for immunotherapy. Future thorough investigations into CCA may provide additional insights into the unexpected effects of existing immunotherapies and foster the development of more effective combination immunotherapies.

**Table 1:** Potential novel therapeutic approaches based on TILs in the management of CCA

**Tumor type**	**Experimental methods**	**Treatment**	**Result**	**References**
ICCA	Animal model	chemokine (C-X-C motif) ligand 9 (CXCL9)	The disruption of natural killer (NK) cell recruitment into the tumor results in an increased tumor burden	[1]
ICCA	Animal model	DNA vaccination targeting CTLA-4/PDL1	The implementation of DNA vaccination directed towards CTLA-4-PDL1 resulted in the inhibition of tumor growth	[2]
ICCA	Animal model	Combined anti-CD40/PD1	The growth of ICCA cells was inhibited, resulting in a longer survival in mice	[3]
CCA	Cell culture experiment	Gemcitabine combined with cytotoxic CTLs	A synergistic effect that enhances the apoptosis of cancer cells	[4]
CCA	Cell culture experiment	Honokiol plus DCs-based vaccine	Honokiol plus DCs-based vaccine enhanced the killing effect of T lymphocytes	[5]
CCA	Cell culture experiment	Cytokine-activated killer (CAK) cells with cetuximab	The co-administration of CAK cells and cetuximab resulted in a substantial increase in cytotoxic activity	[6]

## Abbreviations

B7H4: B7 homolog 4
BTC: biliary tract cancer
CAR T: chimeric antigen receptor T-lymphocytes
CCA: cholangiocarcinoma
CTLA-4: cytotoxic T-lymphocyte associated protein 4
CTLs: cytotoxic T lymphocytes
dCCA: distal cholangiocarcinoma
DCs: dendritic cells
eCCA: extrahepatic cholangiocarcinoma
EGFR: epidermal growth factor receptor
FAS/FASL: Fas cell surface death receptor/Fas ligand
FGFR2: fibroblast growth factor receptor 2
FOXP3: forkhead box protein 3
GemCis: gemcitabine-cisplatin
HHLA2: human endogenous retrovirus-H long terminal repeat- associating protein 2
ICCA: intrahepatic cholangiocarcinoma
ICIs: immune checkpoint inhibitors
IDH1: isocitrate dehydrogenase 1
IL-10: interleukin-10
LAIR2: leukocyte-associated immunoglobulin-like receptor-2
MEOX1: mesenchyme homeobox 1
MHC I/II: major histocompatibility complex class I/II
mIDH1: mutant isocitrate dehydrogenase 1
MUC1: mucin 1
MVD: microvessel density
OS: overall survival
PD1: programmed cell death protein-1
PDL1: programmed cell death ligand-1
PFS: progression-free survival
RECIST: Response Evaluation Criteria in Solid Tumors
scFv: single chain fragment variable
TGF-β1: transforming growth factor beta 1
Th: T helper
TIGIT: T-cell immunoglobulin and ITIM domain
TILs: tumor-infiltrating lymphocytes
TIM3: T cell immunoglobulin and mucin domain-containing protein 3
TME: tumor microenvironment
YAP: Yes-associated protein

## Declarations

### Copyright

© The Author(s) 2023.
